# Femtosecond laser-assisted cataract surgery in a patient with traumatic cataract and corneal opacity after LASIK: a case report

**DOI:** 10.1186/s12886-020-01491-0

**Published:** 2020-06-05

**Authors:** Pei-Wei Huang, Wei-Hsuan Huang, Yuan-Che Tai, Chi-Chin Sun

**Affiliations:** 1 Department of Medicine, College of Medicine, Chang Gung University, Taoyuan City, Taiwan; 2 Department of Ophthalmology, Chang Gung Memorial Hospital, Keelung City, Taiwan; 3 Department of Ophthalmology, Cardinal Tien Hospital, New Taipei City, Taiwan

**Keywords:** FLACS, Cataract, Corneal opacity, LASIK, Case report

## Abstract

**Background:**

Femtosecond laser-assisted cataract surgery (FLACS) has been reported to reduce phacoemulsification time and energy compared to the manual phacoemulsification technique. This technique has been used in several complex cases such as zonular weakness, subluxated lens and traumatic cataracts because it causes less damage to weakened zonules. However, corneal opacity is considered a relative contraindication to FLACS, as it may interfere with laser beam delivery, thus causing unpredictable capsulorhexis and lens fragmentation/liquefaction.

**Case presentation:**

We present here a case with traumatic cataract and corneal opacity after laser-assisted in situ keratomileusis (LASIK). The patient was successfully treated using FLACS, capsular tension ring and intraocular lens (IOL) implantation. Posterior capsule rupture and vitreous loss were noted during the operation. However, the intraocular lens was successfully captured because of a complete capsulorhexis performed by FLACS.

**Conclusion:**

This case report demonstrates that FLACS is a useful tool in selected patients with concurrent corneal opacity and traumatic cataract.

## Key Messages

We reported a case with traumatic cataract and corneal opacity after LASIK who was successfully treated using FLACS. Our report shows that FLACS is a useful tool in selected patients who suffer from cataracts and corneal opacity.

## Background

Femtosecond laser-assisted cataract surgery (FLACS) was first introduced for clinical use in 2009 [1]. This technique is reported to reduce ultrasound energy as compared to the manual phacoemulsification technique [2]. FLACS has been successfully performed in several challenging situations such as corneal penetrating injury [3], lens capsule damage [4], zonular damage [5], and traumatic cataracts [6]. However, corneal opacity remains a relative contraindication to FLACS because it may interfere with the laser beam and might cause anterior capsular tags [7], ineffective lens fragmentation and incomplete corneal wound creation [8] as well. Here, we present a case with traumatic cataract and corneal opacity after LASIK, for whom cataract surgery was performed successfully with the assistance of femtosecond laser.

## Case presentation

**Fig. 1:**
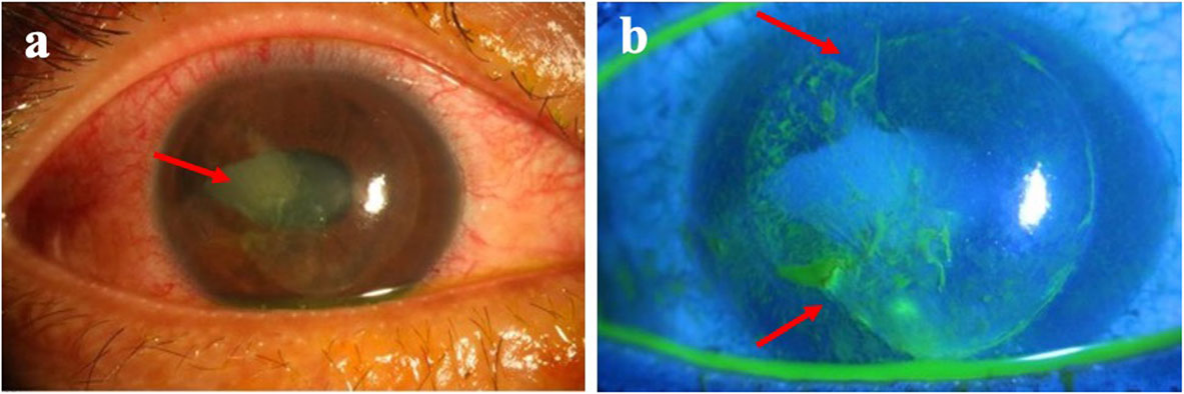
External eye photos of the right eye at the first visit. Superficial corneal opacity (**a**, arrow) and temporal flap loss (**b**, arrows) were noted

**Fig. 2:**
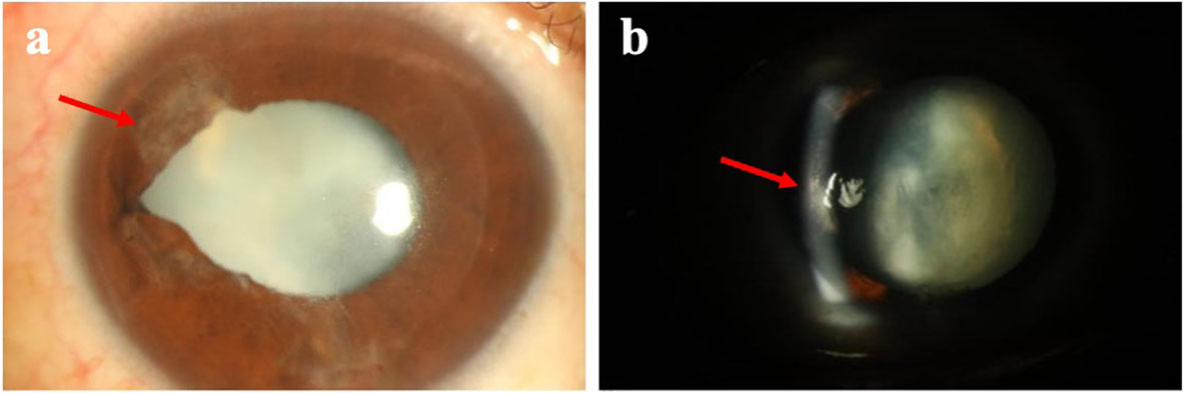
External eye photo of the right eye 5 months after trauma. Progression of traumatic cataract and persisted superficial corneal opacity (arrow) were noted

A 36-year-old man received uneventful bilateral LASIK surgery 6 years prior to sustaining blunt ocular injury to his right eye due to an accident involving cable wires. There was traumatic hyphema and dislocation of the LASIK flap upon injury. Primary repair of the corneal wound in the right eye was performed immediately. Due to epithelial ingrowth, he was referred to our hospital for further management 1 week later. Upon arrival, his uncorrected visual acuity was counting fingers at 30 cm and intraocular pressure was 17 mmHg in the right eye. The LASIK flap was grossly adhered. However, partial LASIK flap loss was noted over the temporal side of the cornea. Moreover, the iris was distorted with vitreous incarceration at 9 o’clock, with appreciation of an extremely deep anterior chamber and traumatic cataract, therefore implying a possibility of a posterior capsular rupture (Fig. 1). Superficial corneal opacity and progression of the traumatic cataract were also noted after healing of the corneal epithelial defect (Fig. 2).

**Fig. 3:**
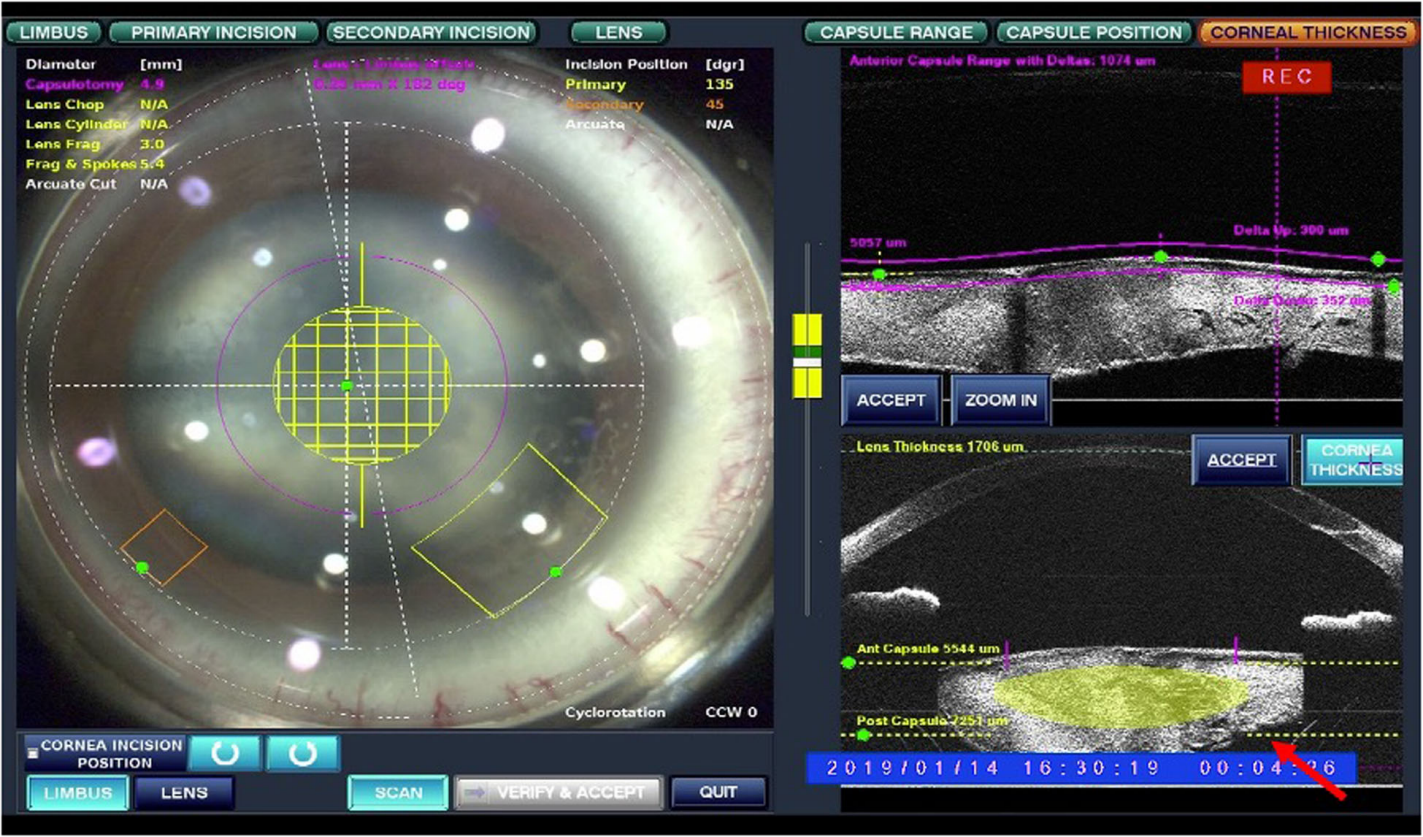
Intraoperative AS-OCT of the right eye. Tilting lens with deep anterior chamber depth and posterior capsule rupture (arrow) were noted

**Fig. 4:**
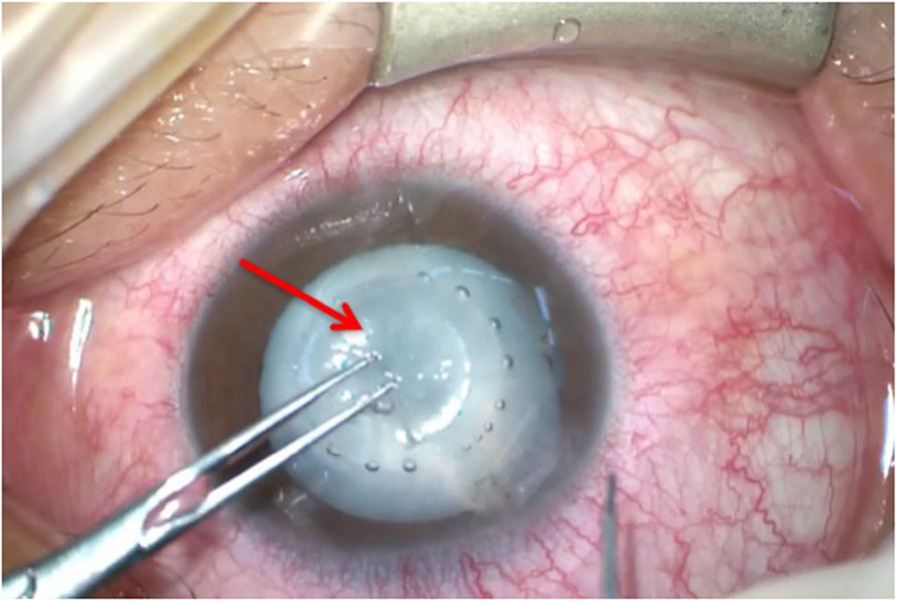
External eye photo of the right eye during operation. The round capsulorhexis (arrow) was completed by femtosecond laser

**Fig. 5:**
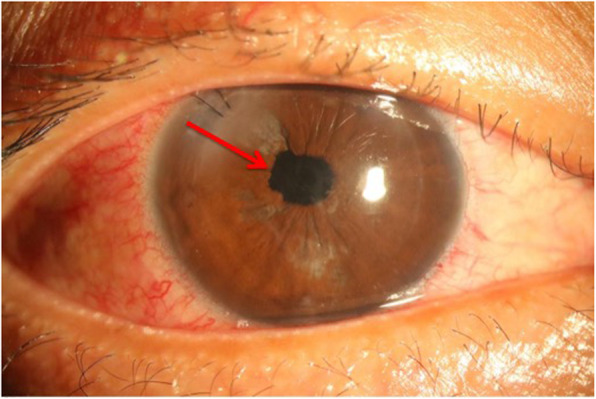
External eye photo of the right eye on post-operative day one. The cataract was successfully replaced by intraocular lens (arrow)

Due to rapid maturation of the cataract in his right eye, the patient received FLACS, capsular tension ring and intraocular lens (IOL) implantation in the sulcus 4months later. The intraoperative anterior segment optical coherence tomography (ASOCT) showed tilted lens, deep anterior chamber depth and tear at the temporal posterior capsule (PC) of his right eye (Fig. 3). Cataract surgery was first performed using the femtosecond laser (Alcon-LenSx Lasers Inc., Aliso Viejo, California, USA). The start and endpoints of the circular cut of the 4.9-mm capsulorrhexis were placed 350 μm behind and 300 μm in front of the anterior capsule, respectively. The lasers also applied to create the main 3.2-mm corneal incision and a 0.9-mm side-port incision. Lens fragmentation was performed using cube pattern with the pulse energy maximized to 10 mJ. During the following conventional phacoemulsification cataract operation, PC rupture and vitreous loss were noted. However, IOL was successfully captured because of a complete capsulorhexis prepared by FLACS (Fig. 4). On postoperative day one, the cataract was successfully replaced by IOL (Fig. 5) and his uncorrected visual acuity improved to 0.6 with the refraction data of − 0.75/− 1.00X110 in the right eye.

## Discussion and conclusion

Cataract surgery is challenging in eyes with corneal opacity because the corneal opacity may limit the visibility of intraocular structures during surgery. In complicated patients with both corneal opacity and traumatic cataracts, the task is even harder since there may be associated zonular weakness or rupture as well as disruption of the lens capsule [5, 6].

FLACS was first used in human eyes in 2009 [1]. With the application of femtosecond laser, three crucial steps of cataract surgery can be refined: positioned self-sealing corneal incisions, well-centered, intact circular capsulorhexis, and lens fragmentation/liquefaction [1]. This technique is also valuable in complicated cases with traumatic cataracts and zonular weakness. Femtosecond laser exerts less tension on the weakened zonules [3, 5] and significantly reduces ultrasound energy when compared to manual phacoemulsification [2]. In addition, the advent of intraoperative ASOCT allows better evaluation of the severity of zonular weakness and the extent of capsule rupture [5]. In this case, intraoperative ASOCT revealed tilting lens, zonular weakness and PC tear. After the application of femtosecond laser, a well-centered capsulorhexis with minimal traction on the zonules and successful lens fragmentation was created. Vitreous prolapse and severe zonular instability were found intra-operatively. There was no lens drop and the IOL was placed in the sulcus with the assistance of a capsular tension ring. In our experience, FLACS is easier and safer for managing traumatic cataracts when compared with manual phacoemulsification.

Corneal opacity is a relative contraindication to FLACS. The delivered laser beam may be disturbed by corneal opacity and cause incomplete capsulorhexis, ineffective lens fragmentation and unpredictable corneal wound creation [8]. There are only a few reports investigating the feasibility of FLACS in patients with traumatic cataracts and corneal opacity. Using customized FLACS, Grewal et al. successfully treated a patient with a traumatic intumescent cataract and midperipheral corneal scar by repositioning the laser delivery zone away from the corneal scar. The best corrected visual acuity improved from hand motion to 20/30 after the operation [9]. Nagy et al. also performed FLACS in a patient who had developed traumatic cataract and peripheral corneal opacity after penetrating trauma by reducing the capsulorhexis size. Post-operatively, the uncorrected visual acuity improved from 20/50 to 20/25 after FLACS [6]. Instead of adjusting the laser delivery zone or reducing capsulorhexis size, the femtosecond laser was directly applied through the corneal opacity in our case. The created capsulorhexis and lens fragmentation were completed by laser treatment. To the best of our knowledge, this report demonstrates for the first time that femtosecond laser can be applied directly through the corneal opacity in a patient with traumatic cataract.

Sometimes, inadequate pupil dilation is a limiting factor for FLACS, especially in cases of concurrent traumatic cataract and iris incarceration. Fortunately, the pupil was well-dilated during the operation in this case after application of topical tropicamide and phenylephrine. It has been reported that pre-operative use of NSAIDs, shorter time lapse between laser application and phacoemulsification can reduce the chance of FLACS-related miosis [10, 11]. With the assistance of a pupil-expanding device, FLACS can also be performed in small-pupil eyes [11].

In conclusion, corneal opacity is generally considered a relative contraindication to FLACS. For this case, we have successfully performed a round, free-floating capsulorhexis and successful lens fragmentation in a patient with corneal opacity and traumatic cataract. However, whether or not FLACS can be used in cases with dense corneal scars or opacities deserves further investigation.

## Data Availability

The datasets used and/or analysed during the current study are available from the corresponding author on reasonable request.

## Abbreviations

LASIK: Laser-assisted in situ keratomileusis
FLACS: Femtosecond laser-assisted cataract surgery
IOL: Intraocular lens
ASOCT: Anterior segment optical coherence tomography
PC: Posterior capsule
